# International medical graduates: challenges and solutions in psychiatry

**DOI:** 10.1192/bji.2021.15

**Published:** 2022-05

**Authors:** Max Pemberton, Sam Nishanth Gnanapragasam, Dinesh Bhugra

**Affiliations:** 1 MRCPsych, Camden and Islington NHS Foundation Trust, London, UK; 2 MRCPsych, Maudsley Hospital, South London and Maudsley NHS Foundation Trust, London, UK. Email: sam.gnanapragasam@slam.nhs.uk; 3 FRCPsych, Institute of Psychiatry, Psychology and Neuroscience, King's College London, UK

**Keywords:** Low and middle income countries, acculturation, healthcare workers, international medical graduates, health workforce

## Abstract

International medical graduates provide a valuable service to the healthcare of their adopted countries. However, there remain a significant number of challenges in their adjustment and acculturation in the post-migration phase. We believe that the cultural capital these doctors bring with them can act as a support as well as a challenge. They are likely to face subtle and not-so-subtle, covert and overt discrimination at a number of levels. In this brief report, we highlight some of the issues faced by them and some potential solutions.

## Background

International medical graduates (IMGs) migrate for a number of reasons and, like all migrants, various push and pull factors may well play a role. People may migrate for educational, political, economic and personal reasons. IMGs are identified as individuals who have graduated from a medical university outside of the country they practise in.^1^ More doctors from outside the UK are joining the workforce than those from within the UK,^2^ although this may change with opening of new medical universities. Between one-third and one-fifth of the medical workforce in countries such as the USA, Canada and Australia are IMGs.^3^ Like all other migrants, IMGs form a heterogeneous group and hail from varied cultural backgrounds. In many instances their exposure to psychiatry in their undergraduate training was limited and their instruction was in a language other than English. And most of the migration, not surprisingly, takes place from low- and middle-income countries to high-income countries. This has implications for the healthcare systems in the countries of origin and has been dubbed a ‘brain drain’, with concerns that this may further global health inequities.

The important components of medical practice include patient care, research and teaching, with occasional forays into management, policy advice, and service design and development. In medical schools around the globe, varied emphasis is placed on these different aspects. This in itself may raise stress levels for newly arrived IMGs, who may not have had any exposure to research, especially related to ethical issues and policy initiatives, and yet may be expected to carry out research or teaching for career progression. Clinical internship after finishing medical school before obtaining full registration has varying components. For example, some countries will insist on a rotation of 3 months each in medicine and surgery, including paediatrics, otolaryngology (‘ENT’) and ophthalmology, obstetrics and gynaecology, and preventive and social medicine, whereas others do 4 months each of medicine, surgery and general practice or something similar, such as psychiatry. These variations mean that IMGs arrive with varying skills, experiences and expectations.

## Challenges

### Training opportunities in donor countries

Around the globe the number of IMGs in psychiatric specialties is impressive and that reflects various factors. In many countries, such as India, the number of training posts in psychiatry is very low and therefore provides local medical graduates limited scope to train in psychiatry. Therefore, some inevitably migrate for professional reasons and may or may not return, owing to a number of factors, such as different healthcare systems. Medical schools on the Indian subcontinent appear to be net exporters of medical workforce, especially to psychiatry.

### Discrimination

Discrimination against migrants is rife in many countries and medical staff are not particularly immune to it. Patients as well as colleagues can carry prejudicial and discriminatory attitudes and behaviours. These negative attitudes related to race, ethnicity, religion, skin colour, language and other factors can be pervasive and make settling down difficult. In the current nationalistic climate in many countries, the atmosphere appears to be getting worse, as are attitudes to IMGs. Beyond just day-to-day experiences of discrimination, there is evidence that IMGs are more likely than local graduates to be reported to the General Medical Council and also more likely to be investigated.^4^ This may be because IMGs are less confident about raising concerns or challenging accusations and are overrepresented in posts more vulnerable to complaints (e.g. locum and more clinically demanding placements).

Another outcome of discrimination is that IMGs end up serving the most socioeconomically disadvantaged, immigrant and minority populations in unpopular geographical locations where local graduates do not wish to settle:^5^ we will return to this below.

### Immigration policies

Flip-flopping of policies on immigration owing to political imperatives often makes it very difficult for IMGs to migrate. It dissuades even the most highly qualified and professionally eager international medical graduates and physicians from even attempting to enter countries that remain in dire need of their services and where they would benefit from training and professional development. In the UK and the USA, the current political environment and rapidly changing immigration policies, which are often very hostile to migrants, will add another layer of complexity, especially when the time comes for the families of IMGs to join them. Countries in which employers have to apply for visas for trainees may be far less attractive to IMGs. Furthermore, it is likely that training schemes will avoid appointing IMGs altogether, as has been seen in the USA, if IMGs need to special visas for residency training.^6^ As Rao and colleagues have cautioned, the effects of the resultant immigration policies will affect not only potential IMG applicants for training but may well influence their career decisions as to whether they stay after completion of their training or return to their countries of origin.^3^

### Cultural capital and acculturation

Every individual, no matter which culture they belong to, carries with them cultural capital (a model used in education). It takes three forms – objective (comprising books, works of arts, music and cultural goods, etc.); embodied (consisting of language, preferences, etc.); and institutional (qualifications, educational and training credentials). Various components of cultural capital vary across cultures and may well contribute to ease or difficulty in the process of acculturation. Of these, institutional culture is perhaps most important to IMGs and yet they may be the most difficult obstacle to overcome. Cultural capital is to be differentiated from economic, social, educational and political capitals, although it may influence these.

Post-migration, IMGs, like other migrants, will attempt to adjust to the norms and mores of the new culture in what has been described as a process of acculturation. Results of acculturation can broadly be assimilation, biculturalism and deculturation. Cultural capital is likely to influence acculturative processes.^7^ Sometimes even when doctors have been trained using the English language, colloquial differences in language can make things unclear and difficult to follow, leading to misunderstandings.

### Differential career progression

There is evidence that in many countries, IMGs reach a glass ceiling. In the UK for example, many IMGs end up as specialty and associate specialist (SAS) doctors. They may also be less likely to be awarded clinical excellence awards and reach positions of power.^8^ This has an impact on pay and contractual rights. And as already mentioned, IMGs tend to end up in less popular subspecialties and unpopular geographical regions.^5^

Interestingly, evidence suggests that where IMGs are given the opportunity, they are able to make significant contributions. For example, according to the Web of Science, 45% of the most cited articles from *JAMA Psychiatry* and the *American Journal of Psychiatry* between 2007 and 2017 had an immigrant or IMG as their senior author.^9^

### Availability of training posts

Until a few years ago, psychiatry's relative lack of popularity with local graduates meant that the specialty used to have many unfilled training positions, which were then filled by IMGs. Another complicating factor has been the impact of Brexit, as IMGs from continental Europe are likely to lose out. Vacancies in training posts can be directly related to immigration policies. What has remained true over the past few decades is that IMG physicians have served in roles historically less preferred by their local counterparts, which is as true in the UK as it is in Canada, the USA and Australia. Polsky and colleagues illustrated from the USA that regions with high poverty rates predicted the practice location of IMG physicians who had emigrated.^10^ Similar observations have been made across the USA, which relies heavily on IMGs to provide their primary mental healthcare^5^ and psychiatric services in rural settings.

It is necessary to acknowledge that, although many are attracted to psychiatry for reasons of clinical interest, some choose this career path because of the low availability of training posts and limited career progression in other specialties. This may be reflected in an IMG's pre-existing exposure to the specialty and skill set development, and more targeted support may be necessary.

## Potential solutions

### Governments

Immigration policies for IMGs and their families ought to be streamlined, with adequate opportunities to undertake work-based employment experiences and observerships. Where changes in immigration policy are made, it must be with sufficient notice and runway in implementation (e.g. more than a year). This is important as those preparing to come to a country often need years to navigate the necessary professional registrations (e.g. language and professional examinations) and visa challenges. Sudden changes in immigration policies therefore jeopardise an IMG's plans at the last stages, leading to a snakes and ladders situation. Further, immigration applications should not be the responsibility of the employers, as this may further add administrative barriers as has been seen in the USA.

### Institutions

Institutions, be they hospitals, universities, regulatory bodies or the UK's medical Royal colleges, have a moral duty to support IMGs. They must set up initial induction courses and follow-up courses for IMGs to learn about their country's healthcare systems, cultural values and differences, linguistic subtleties and nuances in communication. IMGs will need to learn about local legal systems, laws regarding detention and compulsory treatments and community treatment orders where they apply, and also must be taught about assessment of capacity. Regulatory bodies and associations must set up induction in the early days but also follow it up with appropriate sessions on a regular basis. Hospitals, of course, often have their own induction courses and it is crucial that multidisciplinary teams play a part in this, sharing their knowledge and ways of collaborative working. Institutions must also recognise that the training and clinical support necessary will vary in nature depending on whether an IMG gained psychiatric qualifications and training experience abroad or in the UK. For example, support during the examination process, such as with the Clinical Assessment of Skills and Competencies (CASC), may be necessary for those less familiar with such styles of examination. Institutions should also provide representation for IMGs, such as by having an IMG representative on junior and senior doctor committees and boards.

A 20-year analysis of US physicians’ work patterns reported that IMG physicians worked disproportionately more hours in direct patient care and fewer hours in ancillary functions such as administration, medical research and teaching.^11^ In many countries this is beginning to change but it is important that organisations monitor this and ensure that appropriate activities are available depending on an IMG's personal skills and local needs.

### Personal mentoring/peer support

IMGs may need mentoring by their seniors or supervisors to gain support in a semi-formal way with proper support. Peer support groups and Balint groups can also offer support. Schwartz rounds can facilitate multidisciplinary support and learning.

The necessity of adequate and targeted support in light of the significant patient-facing work has been further highlighted in recent months during the COVID-19 pandemic. For example, there has been evidence of the differential demographic and personal risk factors, and the need for personalised risk assessments and tailored work-plans.

## IMGs are an asset to healthcare

As mentioned above, IMG physicians in the USA have been seen to be serving ethnic minority populations at higher rates than local physicians.^11^ US studies also found that Hispanic, Latino, Asian and Pacific Islander patients were twice as likely to visit IMG physicians as a clear preference.^12,13^ It is quite likely that similar patterns exist in other countries. Anecdotally many patients in the UK prefer IMGs because they feel they can talk more easily about spirituality and religious issues. Therefore IMGs carry personal skills and cultural strengths that help the healthcare system provide more culturally appropriate and acceptable care, and may help address challenges related to mental healthcare for marginalised and under-represented populations.

These observations over the past few decades highlight the long-standing critical role played by IMG physicians, who have persisted in serving the neediest sections of the population, in relatively unpopular specialties and unpopular locations while they face ambivalence from immigration authorities and educational discrimination by policymakers.

## Data availability

Data availability is not applicable to this article as no new data were created or analysed in this study.
